# Strategic resource allocation: an agent-based model of ambidextrous strategies in the pharmaceutical industry

**DOI:** 10.1186/s12962-026-00729-w

**Published:** 2026-02-18

**Authors:** Sasan Pourzia, Bahman Hajipour

**Affiliations:** Faculty of Management, Shahid Beheshti University, Tehran, Iran

**Keywords:** Strategic management, Resource allocation, Pharmaceutical strategy, Ambidexterity, Miles and Snow typology, Porter’s five forces, Agent-based modeling, Analytic hierarchy process (AHP)

## Abstract

In this study, we investigate how pharmaceutical firms manage the allocation of their resources between exploitation and exploration based on their competitive standing for the attainment of strategic ambidexterity. The dynamic balance between exploration and exploitation is modeled through iterative agent learning and adaptation, where each firm continuously updates its strategic allocation based on competitors’ performance and market feedback. A data-calibrated agent-based model (ABM) is developed for the simulation of resource-allocation dynamics, incorporating Miles and Snow Typology, Porter’s Five Forces, and Ambidexterity Theory. In distinction to previous studies, which were mostly static and regression-based, our current model is empirically calibrated using data from eight top Iranian firms from 2016 to 2023 and, hence, derives firm behaviors directly out of evidence for behavioral validity and against theoretical bias. Porter’s Five Forces are quantified within the framework of the Analytic Hierarchy Process (AHP), and model robustness is confirmed through calibration via a genetic algorithm and validation by Monte Carlo simulation. Results show that investments in exploration accelerate market-share growth and enhance adaptability. Sensitivity analyses demonstrate the managerial implications of learning rates and observation windows. The findings show how a behavioral estimation-based decision model may connect strategy theory with practical application, thereby constituting an empirically backed framework for adaptive allocation in fast-changing market environments such as the healthcare sector.

## Introduction

The competitive and regulated environment of the pharmaceutical world dictates decisions that increase or decrease the success of longer-term strategic plans for any company. The trade-off between exploration-by investing in new and innovative opportunities-and exploitation-improving efficiencies and utilizing existing capabilities-must be appropriately balanced in order for firms to perform sustainably in future markets [1, 2]. Persistent challenges in ensuring affordability, sustaining innovation incentives, and managing price disparities continue to shape global pharmaceutical pricing policies, constraining firms’ financial stability and access to critical resources [3]. Moreover, the limited effectiveness of economic evaluation studies in informing healthcare resource allocation—especially in low- and middle-income countries, where the methodological flaws and policy misalignments contribute to the muddiness of strategic decision-making—[4] constitute perhaps one of the harshest adversities to decision-making in this regard. This is especially true in Iran, where innovation is confounded by price controls, economic sanctions, prolonged payment cycles, and high costs of R&D [5–7]. The burning question, therefore, is how firms manage to allocate limited resources such that they can pursue both innovation and operational efficiency.

Although the exploration–exploitation trade-off is one of the well-researched areas, almost no study describes how firms dynamically allocate resources between these domains. Most previous works use static or regression-based models that show correlations yet fail to capture feedback-driven strategic adaptation [8–10]. Recent advances, however, prove that data-calibrated agent-based models (ABMs) represent firms’ adaptive behavior better than other methods [11–13]. Their modeling of iterative learning and feedback also reveals how firms continuously revise their strategies. The present study adopts such an approach, inferring adaptive decision rules from real firm data and simulating outcomes that match the empirical market behavior.

Resource Allocation Theory [14], Organizational Ambidexterity [15], and the Miles and Snow Typology [16], all widely studied and well established theories in strategic management, are considered in this study. Resource Allocation Theory accounts for the efficient distribution of resources, but does not indicate how firms resolve the ambidexterity paradox. Ambidexterity Theory indicates this balance but rarely quantifies it [10, 17]. A variation of strategic types-prospector, analyzer, defender, reactor-is established in the subclassification of the Miles and Snow framework; however, it falls short in explicating the role of internal allocation dynamics shaping such classification. Therefore, the association between strategic type and behavior in resource allocation remains underexplored [18].

To bridge this gap, this study creates a data-calibrated ABM that simulates the behavior of pharmaceutical firms in optimizing exploration and exploitation with respect to competitive and regulatory pressures. Porter’s Five Forces are integrated to represent market forces, rivalry, the threat of entry, power of suppliers and buyers, and substitutes about the competitive structure of the industry. This empirical calibration utilizes data from eight leading Iranian firms over the years 2016 to 2023. The work conceptualizes strategy as a dynamic process emerging from adaptive resource allocation, combining computational simulation with real data. By embedding learning and feedback mechanisms into Bower’s theory, it demonstrates how ambidexterity and dynamic capabilities co-evolve in regulated markets. Finally, the study provides a validated quantitative framework for understanding strategic adaptation in complex industries.

## Literature review

The aim of ambidextrous organizations-exploration and exploitation-is one of the premises of the modern strategic management theory [19]. On the one hand, exploration contains innovation and experimentation, while exploitation accounts for efficiency and refinement. The performance of the organization hinges on dynamically balancing and managing the tug of war so that companies do not end up being too heavily or too loosely oriented [20]. This fact becomes even stronger and more striking in the pharmaceutical industry, especially when one takes into consideration that companies here are under great amounts of financial pressure and competition forces with regulations in place against them [21]. The policy instruments such as price controls and reimbursement frameworks in healthcare markets define the financial margins within which firms will navigate macroeconomic and microeconomic decisions [22, 23]. They are not directly shaping behavior but providing incentives and constraints that determine investment and innovation choices.

Research has been assigning cause for strategy to the resource distribution between prevailing competing priorities rather than the organizational structure in which the strategy falls [24]. In this regard, Resource Allocation Theory captures how strategic intent finds its way into investment behavior through the distributed flow of capital, rather than via top-down planning. Previous works have tended to view this process as rational and hierarchical, ignoring the iterative and adaptive processes involved in it [25]. Dynamic Capabilities perspective further extends the rationale in terms of resource reconfiguration as the device for ambidexterity to take place, which would tie strategic intent and adaptive investment behavior [26]. With this in mind, the latest research has discovered that organizational ambidexterity itself arises from resource allocation patterns that entail extensive restructuring by a company of its capital and managerial attention between exploration and exploitation activities necessary for dynamic equilibrium [10].

The Miles and Snow categorization provides an orthogonal lens by classifying firms into prospectors, defenders, analyzers, and reactors, each with distinct orientations toward innovation, efficiency, and adaptability. Prospectors emphasize exploration; defenders emphasize internal efficiency and cost control; analyzers balance the two; reactors respond inconsistently [27]. Prior studies have argued that these archetypes carry the notion of ambidexterity [28], looking at them as behavioral expressions of the balance between exploration and exploitation. However, there still exists a major gap in the understanding of how resource allocations connect to these strategic types. Besides the generality of this typology, the amount of empirical proof to clarify how allocation processes contrast different strategic orientations is very thin [18]. This is a major gap, given that strategy formation has been viewed as an iterative allocation process [14], thus implying that distinct logics of investment, rather than fixed postures, are what characterize each type of strategy.

**Fig. 1:**
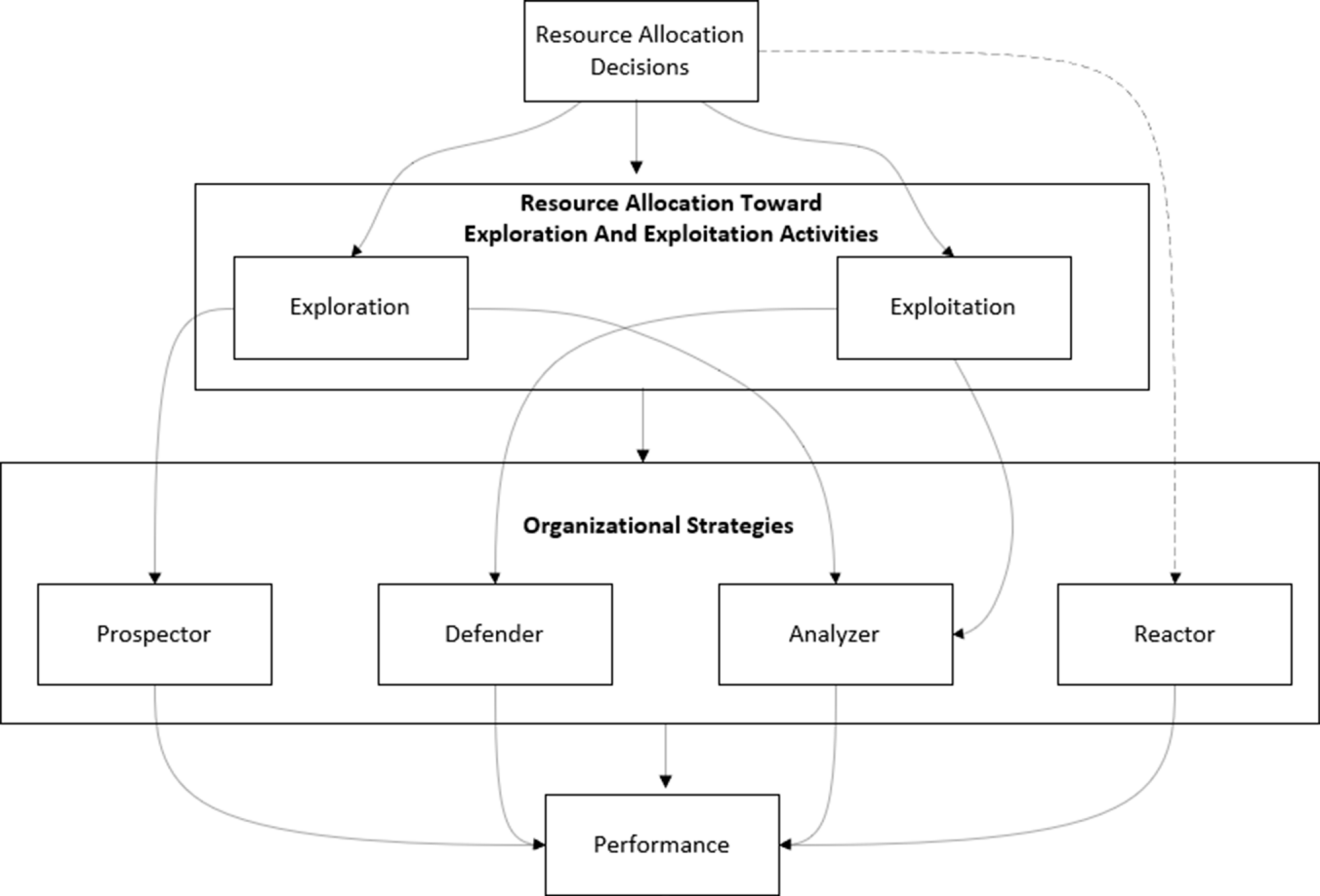
The relationship between firm performance, the miles and snow strategy, ambidexterity, and resource allocation

The study aims to determine how resource allocation strategies correspond with the Miles and Snow archetypes, thus bringing together ambidexterity, resource allocation, and strategic typology into one big framework. The interdependencies among the variables of resource allocation, ambidexterity, strategic archetypes, and performance are shown in Fig. 1. This framework is the basis for a data-calibrated agent-based model (ABM) that simulates how heterogeneous firms adjust their exploration-exploitation ratios through competitive feedback. Specifically, recent developments in behaviorally estimated, data-driven modeling show that these types of approaches are much more capable of capturing the adaptive dynamics than are static or regression-based approaches [29]. In a pharmaceutical context, the evolutionary ABM tried to highlight the impact of policy regimes, patents, and competitive intensity on the pace of innovation and market outcomes [30, 31]. These studies highlight that firm behavior evolves endogenously through feedback and learning, thus reinforcing the behavioral essence of Resource Allocation Theory and showing how adaptive learning shapes performance.

## Methodology

Traditionally, simulation modeling is divided into three paradigms: Discrete-Event Simulation (DES), System Dynamics (SD), and Agent-Based Modeling (ABM). Whereas the first two represent classic modeling approaches, the third gives a large degree of flexibility in modeling dynamic and complex systems [32]. It allows the modeling of non-linearity and time dependence of interactions, thus facilitating the analysis of organizational configurations in their performance, innovation, and resilience [33]. An agent represents an autonomous entity-an independent actor and interactor [34]. This research involves the study of modeled agents: types of Company-Agent (single-agent), Competitor Agents (multi-agent), and Market Agent (single-agent).

### Company agent

Miles and Snow’s strategic typology comprises four types, namely Prospector, Analyzer, Defender, and Reactor, which so far, has become a classic in research on strategic management [18, 27]. In this schema, the strategic behavior of each agent changes concerning the exploration versus exploitation axis. Agents are classified depending on how likely they are to take exploratory as opposed to exploitative actions: Prospectors tend toward exploration, Defenders tend toward exploitation, Analyzers move back and forth between exploration and exploitation well, and Reactors go back and forth between the two or behave rather inconsistently or intermediately. The Analyzer-type agents can adjust their probabilities of the various strategies they should be exploring in an adaptive manner using some kind of learning behavior based on the observed performance of their competitors [28]. Apart from exploration–exploitation probabilities, each agent has its own set of structural and behavioral parameters presented in Table 1.

**Table 1:** The attributes of the company agent

The Company Agent Attribute	Parameter	value
Initial Market share	$MS$	From 0 to 1
Probability to adopt exploitation strategy	${P_{exploit}}$	From 0 to 1
Probability to adopt exploration strategy	${P_{explore}}$	From 0 to 1
Efficiency of the company in implementing an exploitation Strategy	$E{F_{exploitation}}$	From 0 to 1
Efficiency of the company in implementing an exploration Strategy	$E{F_{explore}}$	From 0 to 1
Market share gained through successful implementation of the exploitation/exploration strategy	$rewar{d_{exploit/explore}}$	From 0 to 1
market share lost due to unsuccessful implementation of the exploitation/exploration strategy	$penalt{y_{exploit/explore}}$	From 0 to 1
Learning rate for leveraging the experience	$\alpha$	From 0 to 1
Duration between leveraging the experience of other companies	D	From 0 to N
Number of companies selected for leveraging the experience	N	
Level of awareness about competitors’ performance	$A{w_{min - max}}$	Discrete uniform(min number of agents, max number of agents)
Max number of exploitation adoptions	${N_{exploit}}$	From 0 to N
Max number of exploration adoptions	${N_{explore}}$	From 0 to N

The following expressions will hold regarding the attributes of the agents:

1 $${P_{exploit}} + {P_{explore}} = 1$$

2 $$rewar{d_{\,explore}} \ge rewar{d_{\,exploit}}$$

3 $$penalt{y_{\,explore}} \ge penalt{y_{\,exploit}}$$

**Fig. 2:**
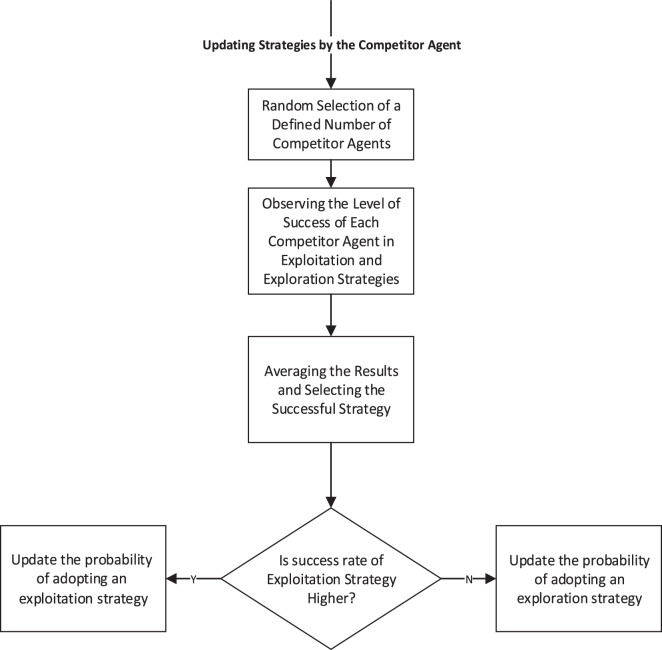
Logic of updating strategy adoption by leveraging the experience of other agents

When an agent allocates resources to a particular strategy, it gains a market share of $rewar{d_{exploit/explore}}$ upon success. Conversely, if the strategy fails, the agent’s organization experiences a loss of market share equal to $penalt{y_{exploit/explore}}$, firms continuously revise their exploration–exploitation allocations by learning from competitors and market signals. According to the model, before which such updating probabilities are at defined intervals (D) in the advent of competitors’ observed performance, consistent with bounded rationality [35] and newer developments in agent-based modelling of strategic competition [36, 37], each agent is taken to possess limited awareness of the environment, evaluating only a number of competitors before modifying its allocation according to a learning parameter α. Figure 2 illustrates this updating mechanism.

To explicitly operationalize the temporal dynamics of ambidexterity, the model implements firms’ learning and strategy-updating behavior through the following step-by-step procedure:

1. Initialization: At the beginning of the simulation, each company is assigned two initial probabilities, $p_t^{explore}$ and $p_t^{exploit}$, representing the firm’s tendency to adopt exploration and exploitation strategies, respectively. These initial values are calibrated from empirical evidence (interviews and historical strategic patterns).
2. Strategy selection: During each simulation period, the firm chooses whether to pursue exploration or exploitation probabilistically according to its current probabilities $p_t^{exploit}$ and $p_t^{explore}$.
3. Outcome realization: The chosen strategy leads to a success or failure outcome (determined by the market-agent success probability and the firm’s implementation efficiency).
4. Scheduled learning moments (every $D$ periods): Firms do not update their strategic orientation continuously. Instead, they revise their probabilities only at predefined review intervals, every $D$ periods, capturing bounded rationality and periodic managerial evaluation cycles.
5. Observing and updating behaviors: At every $D$ period interval, the firm samples a few competitors and compares the success of exploration and exploitation activities observed among them. Based on the competitors’ most successful strategic route, the firm’s own strategic orientation is reinforced proportionately according to the following rule:
  $$\left( {p_{t + 1}^{exploit},p_{t + 1}^{explore}} \right) = \left\{ {\begin{array}{*{20}{c}} {p_t^{exploit}\left( {1 + \alpha } \right),\,p_t^{explore} - \alpha p_t^{exploit},{\mathrm{if}\,}\,{E_t} > {R_t}\left( 4 \right)} \\ {p_t^{exploit} - \alpha p_t^{explore},\,p_t^{explore}\left( {1 + \alpha } \right),{\mathrm{if}}\,{E_t} < {R_t}\left( 5 \right)} \\ {p_t^{exploit},\,p_t^{explore},{\mathrm{otherwise}}\left( 6 \right)} \end{array}} \right.$$
  where $\alpha$ is defined as the company’s learning rate while ${E_t}{\rm{ }}$ and ${R_t}{\rm{ }}$ are the number of times exploitation and exploration strategies have been observed to be successful among the sampled competitors, respectively.
6. Normalization: After updating, $$p_{t + 1}^{exploit} and p_{t + 1}^{explore} $$are normalized after each update to maintain their probabilistic consistency, keeping their sum equal to one.

This procedure captures dynamic managerial attitudes away from the less successful strategy noted in the market toward the more successful one while allowing for some emergent dynamic balance between exploration and exploitation, all without disturbing the initial model structure.

In this study, one of the company agents is designated as the primary focus for performance analysis, referred to as the “Company Agent.” The remaining agents function as competitor agents within the simulation model.

### Market agent

In this study, Porter’s Five Forces framework is applied to capture the structural dynamics governing competition in the Iranian pharmaceutical industry. Industry structure—defined by rivalry among existing firms, threat of entry, bargaining power of buyers and suppliers, and threat of substitutes—is shaped within a broader institutional and macroeconomic context that includes regulation, price controls, sanctions, and technological conditions [38]. Following Porter’s theoretical logic, such contextual factors are not treated as independent competitive forces; rather, they function as institutional moderators that strengthen or weaken the intensity and interaction of the five forces already present. Accordingly, while the model explicitly operationalizes Porter’s five forces, government policies and similar constraints are implicitly incorporated through their systematic influence on competitive intensity in a regulated market environment. This approach ensures conceptual consistency between real market conditions and the analytical structure of the model [39].

Within the simulation, the Market Agent represents this competitive environment. Modeled as a single-agent system, it determines the success probabilities of exploration and exploitation strategies based on overall market competitiveness. These dynamics are quantified through Porter’s framework, which links the five forces affecting innovation and efficiency of firms: threat of new entrants (TONE), bargaining power of suppliers (BPOS), bargaining power of buyers (BPOB), threat of substitutes (TOS), and intensity of rivalry (IR). The attributes of the Market Agent are summarized in Table 2.

**Table 2:** The attributes of the market agent

The Market Agent Attribute	Parameter	value
Market score for threats of new entrants	TONE	From 1 to 9
Importance weight of TONE in exploit strategy success	${W_{TONE - exploit}}$	From 0 to 1
Importance weight of TONE in explore strategy success	${W_{TONE - explore}}$	From 0 to 1
Market Score for bargaining power of suppliers	BPOS	From 1 to 9
Importance weight of (BPOS) in exploit strategy Success	${W_{BPOS - exploit}}$	From 0 to 1
Importance weight of (BPOS) in explore strategy Success	${W_{BPOS - explore}}$	From 0 to 1
Market score for bargaining power of buyers	BPOB	From 1 to 9
Importance weight of BPOB in exploit strategy Success	${W_{BPOB - exploit}}$	From 0 to 1
Importance weight of BPOB in explore strategy Success	${W_{BPOB - explore}}$	From 0 to 1
Market score for threat of substitutes	TOS	From 1 to 9
Importance weight of TOS in exploit strategy Success	${W_{TOS - exploit}}$	From 0 to 1
Importance weight of TOS in explore strategy Success	${W_{TOS - explore}}$	From 0 to 1
Market score for intensity rivalry	IR	From 1 to 9
Importance weights of (IR) in exploit strategy Success	${W_{IR - exploit}}$	From 0 to 1
Importance weights of (IR) in explore strategy Success	${W_{IR - explore}}$	From 0 to 1

The following expressions apply to the Market Agent’s attributes:

7 $$\begin{gathered}{W_{TONE - exploit}} + {W_{BPOS - exploit}} + {W_{BPOB - exploit}} \hfill \\+ {W_{TOS - exploit}} + {W_{IR - exploit}} = 1 \hfill \\\end{gathered} $$

8 $$\begin{gathered}{W_{TONE - explore}} + {W_{BPOS - explore}} + {W_{BPOB - explore}} \hfill \\+ {W_{TOS - explore}} + {W_{IR - explore}} = 1 \hfill \\\end{gathered} $$

By means of the AHP (Analytic Hierarchy Process) for the pairwise comparison, this research quantified the influences of Porter’s Five Forces on the success of exploration and exploitation strategies. The opinions of experts were fed into a structured rating process in which judgments were captured along a nine-point Saaty scale for expressing the relative importance of each force. After determining the importance weight of each force for both strategies, the respective market situations were evaluated, in terms of being assigned a score representing how well each force corresponded to the market. The overall probability is then computed according to the following expression:

9 $${\mathbf{SuccessProb}} = 1 - \frac{\begin{gathered}\left( {{\mathrm{TONE}} \times {W_{TONE}}} \right) + \left( {{\mathrm{BPOS}} \times {W_{BPOS}}} \right) + \hfill \\\left( {{\mathrm{BPOB}} \times {W_{BPOB}}} \right) + \left( {{\mathrm{TOS}} \times {W_{TOS}}} \right) + \left( {{\mathrm{IR}} \times {W_{IR}}} \right) \hfill \\\end{gathered} }{9}$$

Since all of Porter’s Five Forces are conceptualized as competitive pressures, higher force intensities indicate stronger competitive constraints and, consequently, a lower likelihood of strategic success. Accordingly, the complement of the aggregated force-based probability is used to represent the probability of success. It should be emphasized that this formulation does not imply that the five forces are inherently negative; rather, consistent with Porter’s framework, they capture the structural intensity of competition. Opportunities therefore arise endogenously when these pressures weaken, reflecting more favorable industry conditions rather than a reversal of force effects [40]. In addition, an efficiency factor is assigned to each agent to capture firm-specific capability in implementing exploration or exploitation strategies. Consequently, strategic success is determined jointly by market structure and firm-level execution ability. The final calculation of success probability for each strategy is therefore given as:

10 $$\begin{gathered}{\mathbf{Exploit}}\,{\mathbf{success}}\,{\mathbf{probability}}\,{\mathbf{for}}\,{\mathbf{the}}\,{\mathbf{agent}} = \hfill \\{\mathbf{exploit}}\,{\mathbf{Success}}\,{\mathbf{Prob}}*E{F_{exploit}} \hfill \\\end{gathered} $$

11 $$\begin{gathered}{\mathbf{Explore}}\,{\mathbf{success}}\,{\mathbf{probability}}\,{\mathbf{for}}\,{\mathbf{the}}\,{\mathbf{agent}} = \hfill \\{\mathbf{explore}}\,{\mathbf{Success}}\,{\mathbf{Prob}}*E{F_{explore}} \hfill \\\end{gathered} $$

**Fig. 3:**
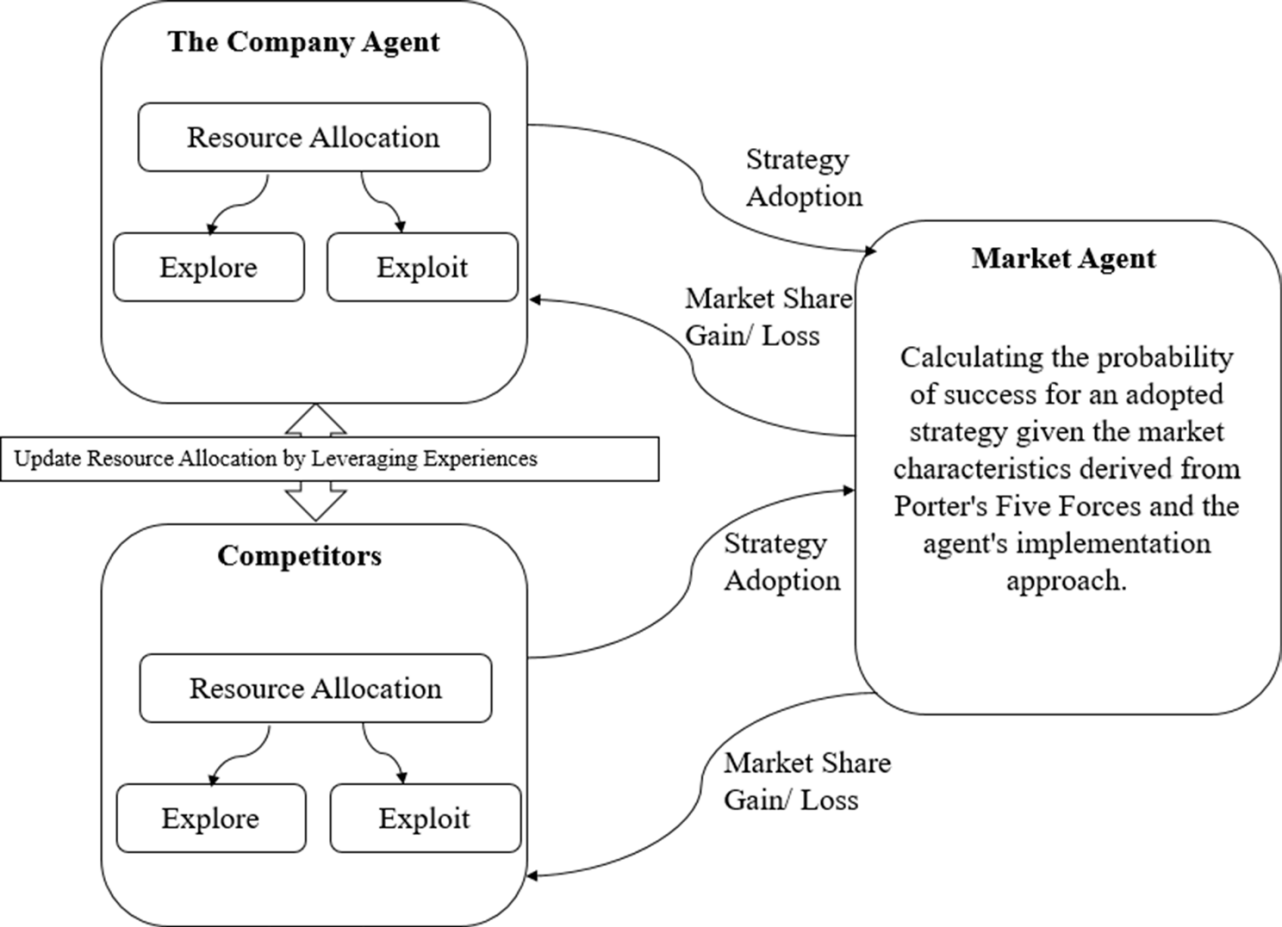
Conceptual agent-based model

Under bounded rationality, agents infer strategy outcomes from market share changes rather than directly observing success probabilities (Manson, 2006). The conceptual model is shown in Fig. 3.

## Empirical analysis

This empirical section is based on calibration and validation. It will detail the characteristics of the sampled firms, data sources, and methods of parameter estimation that connect our model to actual market behavior.

## Case study

With slow reimbursement cycles, exchange rate fluctuation, and high dependence on imported raw materials, firms in the Iranian pharmaceutical sector face difficulties in conducting R&D and making capital investments [7, 41]. Other variables accentuating the severe conditions of liquidity and supply-chain costs are price controls, delayed payments from insurance companies, and international sanctions [42, 43]. The activities in some strategic areas presented in Table 3 are the result of extensive interviews with industry experts and a detailed literature review. This classification, therefore, uses definitions provided in existing literature, ensuring conceptual alignment with recognized strategic constructs.

**Table 3:** Exploitation and exploration strategies in the pharmaceutical market

Exploration Strategies	Exploitation Strategies
Development of a First-in-Class Drug	Extended-Release Formulation
New Drug for an Unmet Medical Need	Fixed-Dose Combination
Discovery and Launch of a New Drug Class	injectable to oral form or vice versa
Exploring New Therapeutic Areas	New strength or dosing options
Pioneering a New Treatment Modality	Improving Drug Delivery Systems
Creating a Drug for a Rare Disease	Label Expansion
Exploring New Indications for a Novel Drug	Pediatric or Geriatric Formulations
Introducing a New Drug in a Competitive Market	Rebranding Existing Drugs

With more than 300 active firms in the Iranian pharmaceutical market, this study focuses on eight leading companies that collectively account for approximately 30% of national sales. The selection followed a homogeneous sampling logic, whereby firms were intentionally drawn from the same structural size category—very large pharmaceutical firms, defined as companies belonging to the upper quartile of national pharmaceutical sales, with sustained nationwide distribution, diversified multi-therapeutic portfolios, and formalized organizational structures for R&D and strategic decision-making. This approach was adopted to control for substantial heterogeneity in resource availability, innovation capacity, and governance structures that characterizes the Iranian pharmaceutical industry and could otherwise confound strategic dynamics [44, 45].

Within this homogeneous group, firms were further selected based on criteria designed to minimize sampling bias and maintain theoretical relevance: (1) financial transparency, defined as the availability of continuous audited statements on Codal; (2) a broad and diversified product portfolio across major therapeutic classes, enabling comparability of strategic choices; and (3) verified engagement in both exploration and exploitation activities, assessed through pre-screening interviews with CEOs, R&D directors, and business-development managers. Methodological guidance for multiple-case designs indicates that four to ten analytically relevant cases are typically sufficient for cross-case comparison and analytic generalization [46]; the inclusion of eight firms falls within this established range. These criteria collectively provide a well-documented and analytically relevant basis for empirical calibration. Data for model calibration were obtained from different sources:

- Financial statements spanning the past 8 years [47].
- Interviews with industry experts and surveys.
- Online Pharmaceutical Statistics System [48, 49].
- Sales and product data acquired from pharmaceutical websites.

A set of semi-structured interviews with 24 managers (approximately three from each company), including CEOs, R&D directors, and business development managers. The questionnaire was made up of three parts: (1) strategic orientation and practices of innovation, (2) resource allocation between exploration-exploitation, and (3) perceived competitive and regulatory forces according to Porter’s Five Forces. Reliability and validity were verified through cross-analysis with the Codal and FDA datasets for the first two sections. In the case of the third section, pairwise comparisons of Porter’s forces were completed by participants and all of them resulted in consistency ratios (CR) below 0.10 indicating reasonable logical coherence. The internal reliability of qualitative data was confirmed furthermore by Cronbach’s alpha (α = 0.87). To keep anonymity, companies are cited by identifying them as Company 1–8, while their financial information is depicted in summary in Table 4.

**Table 4:** Yearly income of the companies (billion rials)

	2016	2017	2018	2019	2020	2021	2022	2023
Company 1	3,064.7	4,040.1	6,058.4	9,484.8	14,936.8	24,910.0	45,216.3	70,517.9
Company 2	1,800.8	2,385.9	3,682.1	5,525.9	8,882.3	14,870.5	28,007.0	42,417.3
Company 3	911.1	1,246.5	1,949.0	3,020.9	4,258.7	4,258.7	13,596.8	21,268.4
Company 4	160.3	233.7	395.9	602.1	899.5	1,410.0	2,885.7	3,614.3
Company 5	2,992.5	3,999.1	5,866.9	9,721.7	14,526.7	23,970.0	46,703.2	72,434.6
Company 6	2,805.5	3,464.7	4,978.5	8,020.3	11,690.5	25,478.3	42,120.0	62,100.0
Company 7	3,029.2	3,609.4	5,176.1	7,451.5	8,989.7	14,610.1	23,214.7	31,603.0
Company 8	1,557.9	1,914.7	2,718.8	3,872.1	5,394.4	7,251.1	11,700.0	17,250.0

To provide a comprehensive overview of the pharmaceutical market, Table 5 summarizes the overall revenue status of pharmaceutical companies, including the eight primary players.

**Table 5:** Total pharmaceutical market value (billion rials)

Total Market	2016	2017	2018	2019	2020	2021	2022	2023
Value (Billion Rials)	73,828.3	91,177.6	131,981.4	200,697.7	283,913.4	470,000.0	780,000.0	1,150,000.0

In classifying the eight selected pharmaceutical companies, in-depth analysis on strategic activities for each firm was carried out through information obtained from interviews and sales data. Each company was thus categorized according to specific attributes based on their strategic inclinations into one group in lieu of the Miles and Snow typology. For instance, the percentages of exploitation strategy adoption ${P_{exploit}}$ and exploration strategy adoption ${P_{explore}}$ which had been derived through interviews with the firm’s CEOs, Research and Development directors, and business development managers are indicated in Table 6.

**Table 6:** Grouping of companies based on Miles and snow typology and probability of strategy adoption

	Type	${P_{exploit}}$	${P_{explore}}$
Company 1	Prospector	10%	90%
Company 2	Analyzer	43%	57%
Company 3	Analyzer	33%	66%
Company 4	Analyzer	63%	37%
Company 5	Analyzer	56%	44%
Company 6	Analyzer	33%	66%
Company 7	Defender	89%	11%
Company 8	Defender	75%	25%

A threshold of 30% was used to identify the predominant strategic orientation of each of the firms. In the simulation model, however, the companies act probabilistically according to their rates of exploration and exploitation; whereas for the purpose of classification according to the Miles and Snow typology, firms were thus classified according to the relative dominance of those probabilities. In other words, if a company has an exploration probability ${P_{explore}}$ is equal to or greater than 70%, it is categorized among Prospectors as otherwise classified by those with an exploitation probability ${P_{exploit}}$ equal to or higher than 70%, while the companies in between these lines will be classified as Analyzers.

The Analytic Hierarchy Process pairwise comparison method was used to assess the impact of Porter’s Five Forces on both strategic orientations; the method has been well established during the competitive dynamics assessment [50–52]. Experienced experts in pharmaceutical strategies performed pairwise comparisons ranking the five forces for exploration and exploitation contexts as to their relative importance. The qualitative judgments were then quantified using a nine-point Saaty scale to generate normalized weights for each force under both strategies. During the expert evaluation process, participants were explicitly instructed to consider regulatory and policy conditions of the Iranian pharmaceutical market, including price controls, reimbursement delays, and international sanctions, when scoring and weighting the five competitive forces. As a result, these institutional factors are reflected in the empirical inputs used for model calibration. The computed weights are provided in Table 7.

**Table 7:** Index weights in exploitation and exploration strategies

Index	Weight in Exploitation Strategy	Weight in Exploration Strategy
Threat of New Entrants	33.3%	28.4%
Bargaining Power of Customers	16.7%	12.1%
Bargaining Power of Suppliers	12.5%	12.5%
Threat of Substitutes	20.8%	24.9%
Intensity of Rivalry	16.7%	22.1%
Total	100%	100%

After having derived index weights through AHP pairwise comparison, each one of Porter’s Five Forces in the Iranian pharmaceutical market was evaluated on a nine-point Likert scale, where 1 means very low and 9 high. A group of experts in the field of pharmaceuticals rated the forces and averaged out the scores with equal weight, thus allowing a level playing field in comparable expertise and experience levels. The resulting mean ratings are shown in Table 8, giving an idea of the relative strength of each force within the Iranian context for pharmaceutical products.

**Table 8:** Likert scale score for the pharmaceutical market in Iran

Index	Likert Scale Score
Threat of New Entrants	3.3
Bargaining Power of Customers	1.4
Bargaining Power of Suppliers	5.2
Threat of Substitutes	5.8
Intensity of Rivalry	6.6

Finally, based on Eq. (9), the success probability of agents in adopting exploitation strategies is calculated to be 52.5%, while for exploration strategies, it is 48.2%.

## Agent-based model calibration and validation

Once the agent-based simulation is built, a major task consists of validating it against the real dynamics of the pharmaceutical market. Weights are assigned to model calibration so that the model behaves like the real observations. Candidates for the calibration coefficients have thus been:

1. $C{S_{exploit}}$: Market share increase coefficient after the successful implementation of the exploitation strategy
2. $C{F_{exploit}}$: Market share decrease coefficient after the unsuccessful implementation of the exploitation strategy
3. $C{S_{explore}}$: Market share increase coefficient after the successful implementation of the exploration strategy
4. $C{F_{explore}}$: Market share decrease coefficient after the unsuccessful implementation of the exploration strategy
5. $P{C_{explore}}$: Company performance coefficient in exploration strategy adoption
6. $P{C_{exploit}}$: Company performance coefficient in exploitation strategy adoption

The main goal of the process of calibration was to reduce the difference between the actual market share of the company from 2016 until 2023 and the one estimated through the agent-based simulation model during the same time period. A Genetic Algorithm has been used for optimization of an objective function consisting of the following components:

**Objective function**: The objective function aims to minimize the sum of squared errors (SSE) between the actual and simulated market shares for the company during the 2016–2023 period. Mathematically, it can be expressed as:

$$\begin{gathered}MinZ = \hfill \\\sum\limits_{y = 2016}^{2023} {\left| {Market\,Share\,Rea{l_y} - Market\,Share\,Simulatio{n_y}} \right|} \hfill \\\end{gathered} $$

**Decision variables**: The decision variables for the GA optimization include the six calibration coefficients previously mentioned.

The calibration process involved an initial 4000 iterations for the calibration of model coefficients for each of the eight companies in the pharmaceutical sector. Given the stochastic nature of the agent-based simulation model, each iteration was replicated 1000 times to ensure convergence and stability of the results. The number of iterations was derived from the observation that there were no major improvements in the objective function during the calibration for all companies. Results of the calibration process are provided in Table 9.

**Table 9:** Calibration process results of the agent-based model

	Company 1	Company 2	Company 3	Company 4	Company 5	Company 6	Company 7	Company 8
$C{S_{exploit}}$	0.072	0.088	0.037	0.094	0.145	0.166	0.141	0.151
$C{F_{exploit}}$	0.022	0.02	0.011	0.03	0.061	0.1	0.1	0.105
$C{S_{explore}}$	0.157	0.13	0.071	0.159	0.267	0.282	0.174	0.307
$C{F_{explore}}$	0.044	0.062	0.042	0.061	0.151	0.167	0.131	0.202
$P{C_{explore}}$	1.05	1.12	0.883	0.998	1.15	1.01	0.211	0.228
$P{C_{exploit}}$	1.009	1.05	1.02	0.971	1.143	0.98	0.203	0.195

**Fig. 4:**
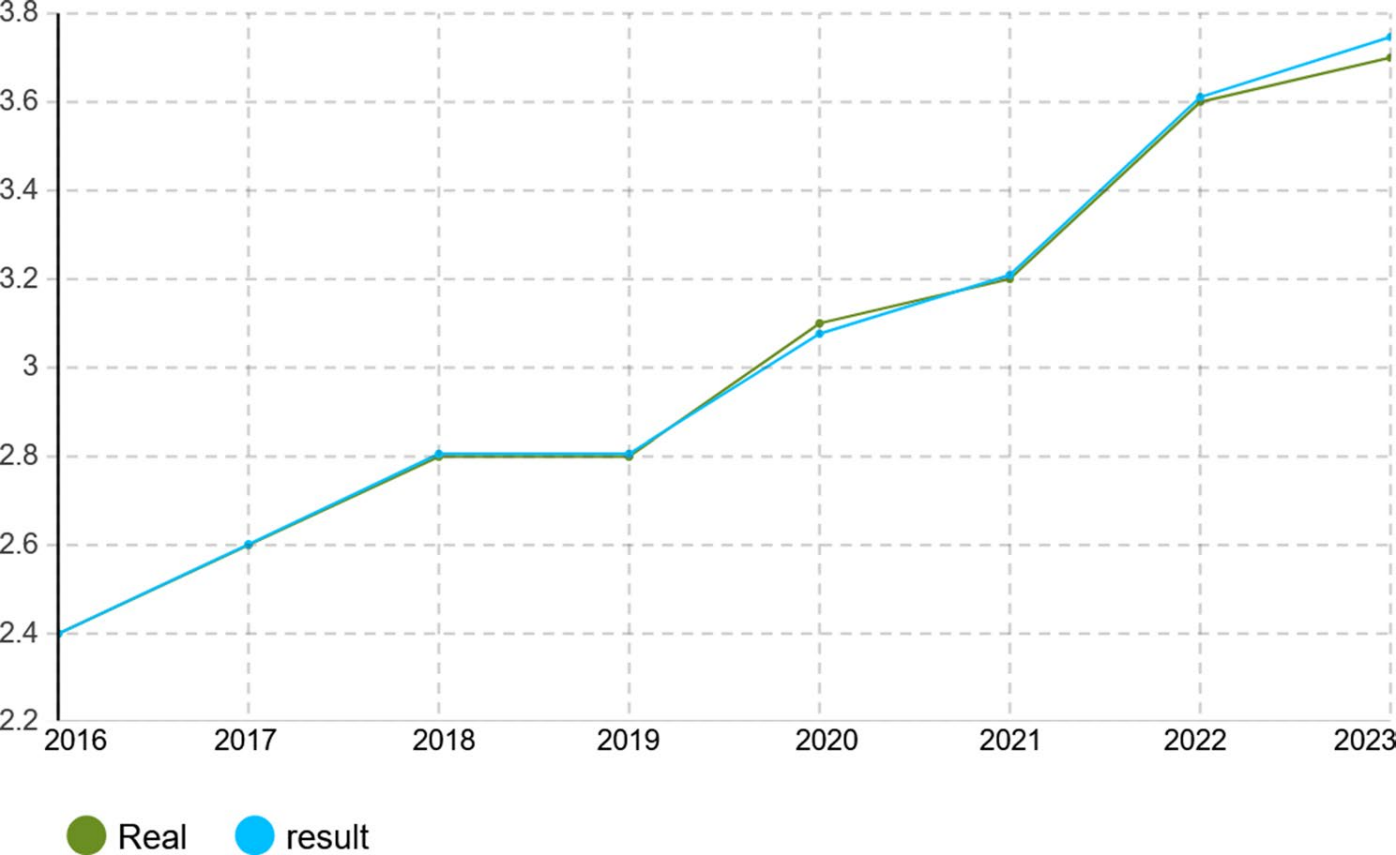
Comparison of actual vs. Simulated market share for company 1

**Fig. 5:**
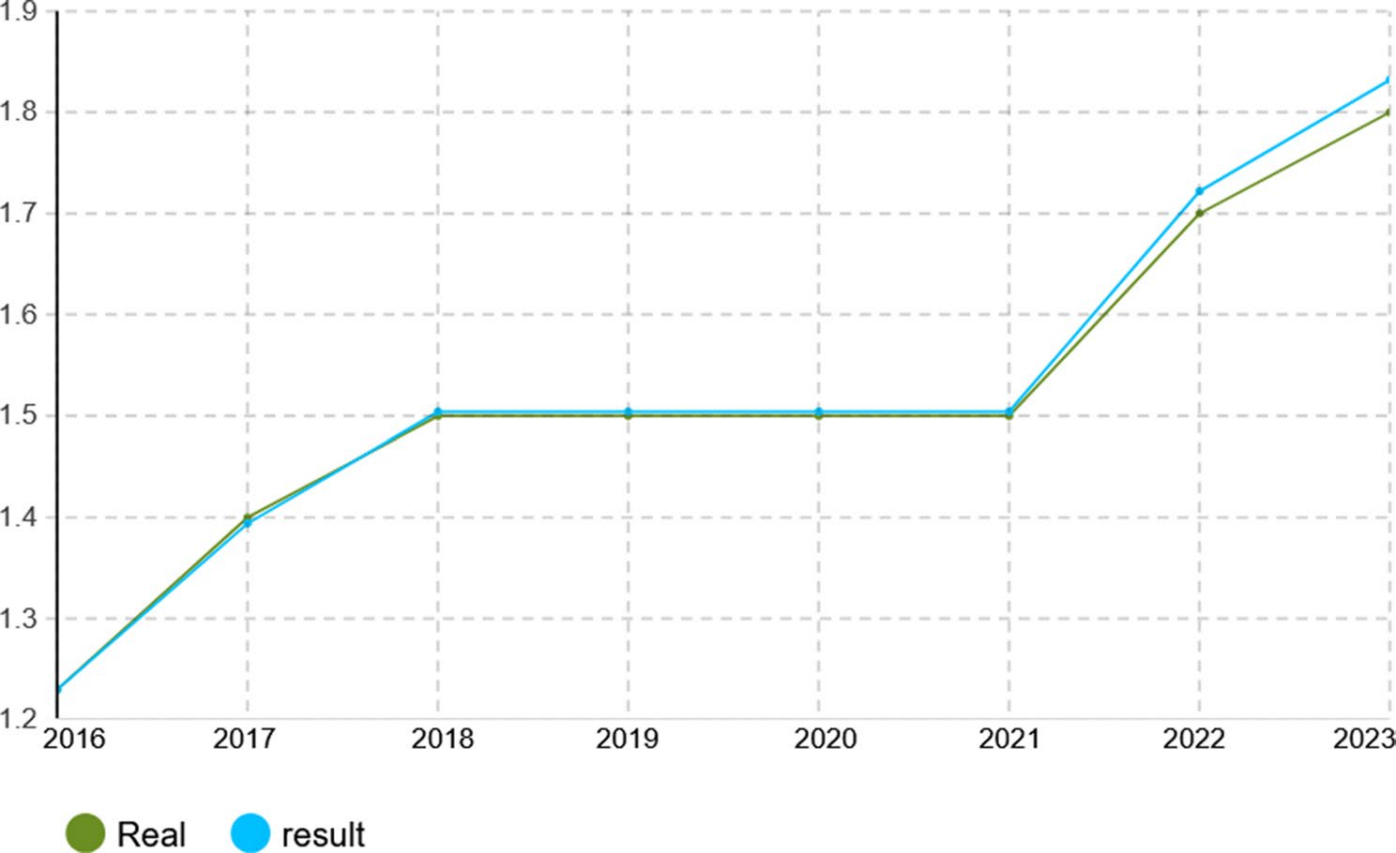
Comparison of actual vs. Simulated market share for company 2

**Fig. 6:**
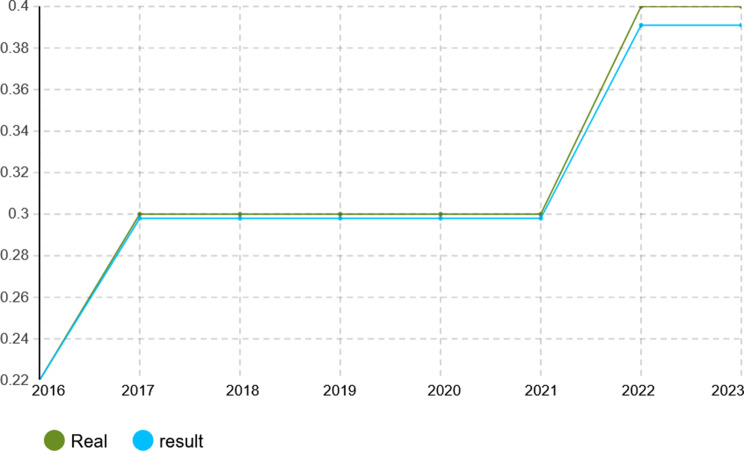
Comparison of actual vs. Simulated market share for company 3

**Fig. 7:**
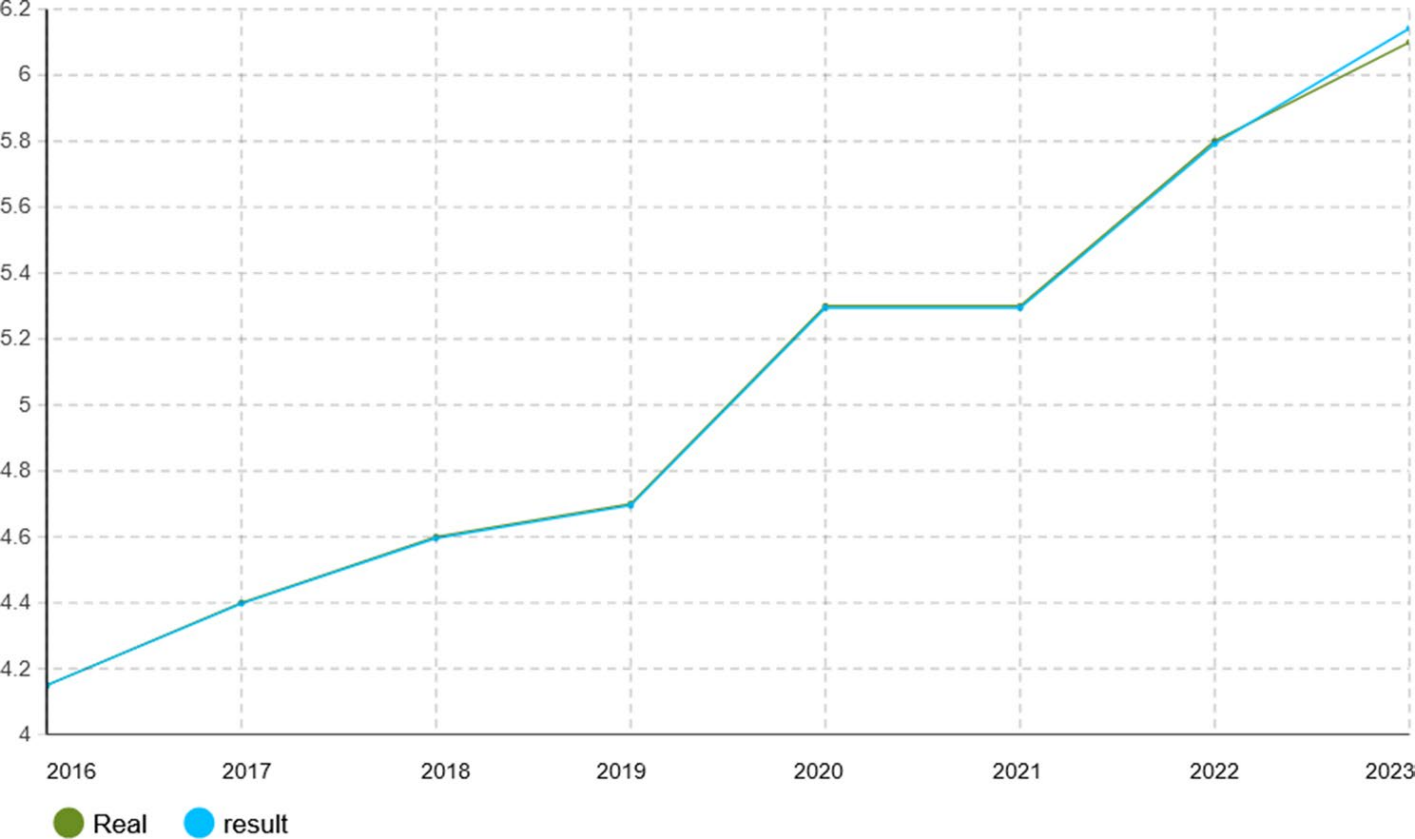
Comparison of actual vs. Simulated market share for company 4

**Fig. 8:**
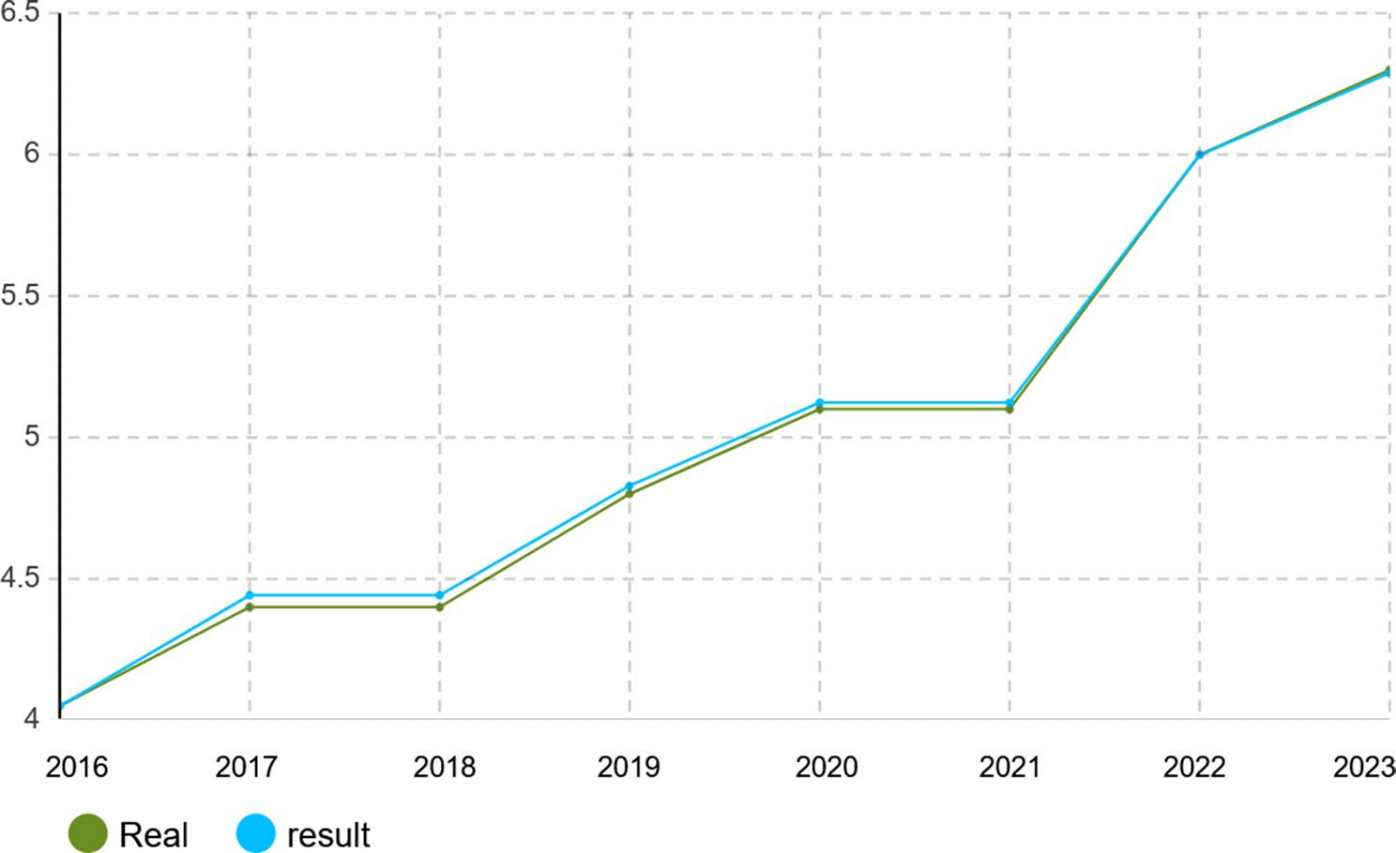
Comparison of actual vs. Simulated market share for company 5

**Fig. 9:**
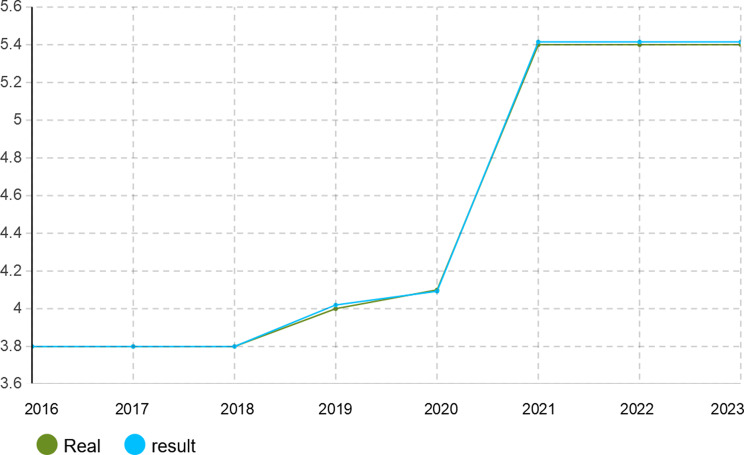
Comparison of actual vs. Simulated market share for company 6

**Fig. 10:**
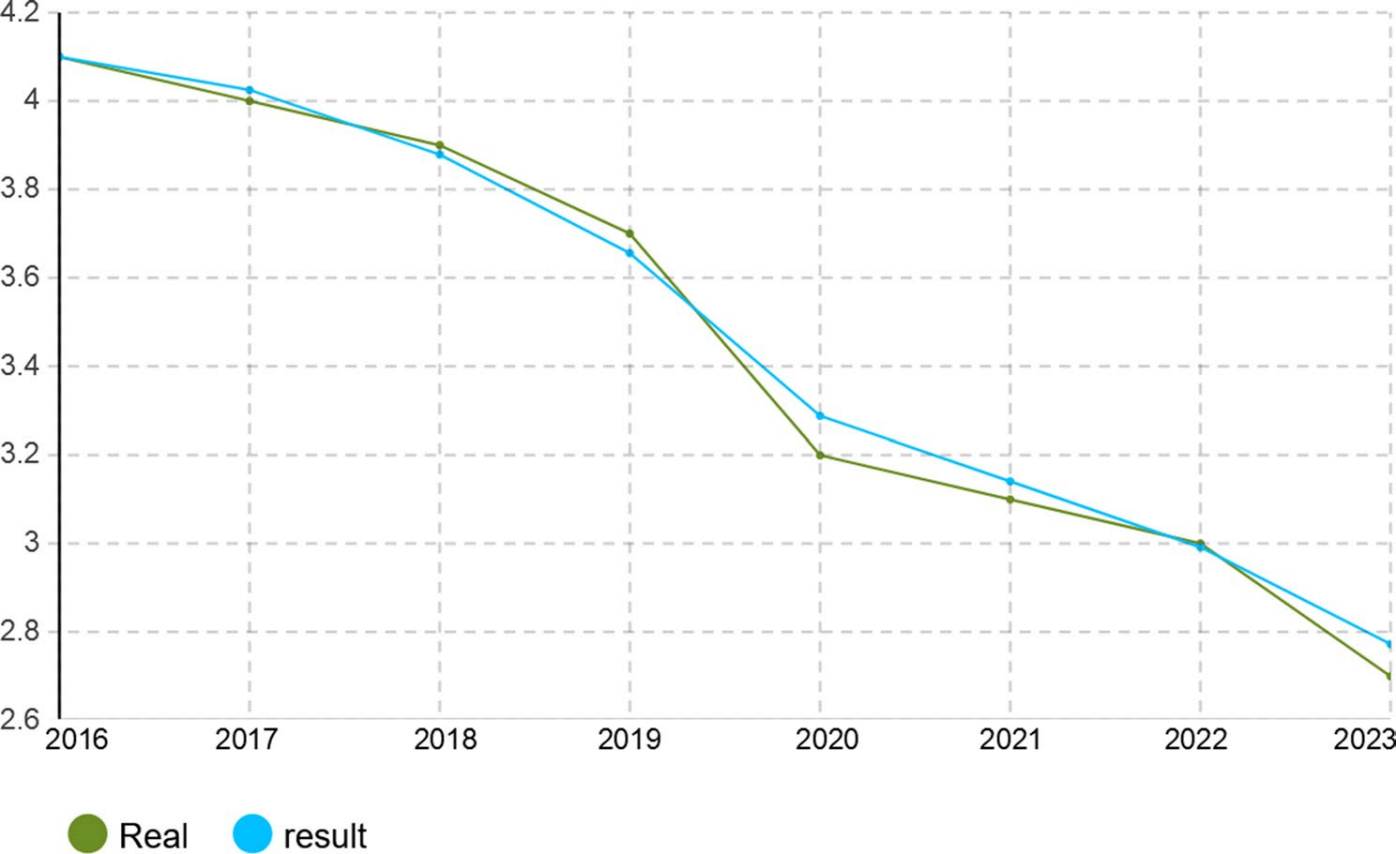
Comparison of actual vs. Simulated market share for company 7

**Fig. 11:**
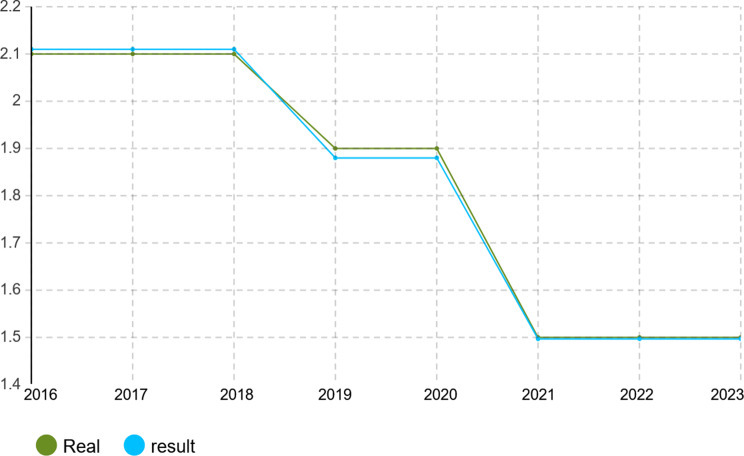
Comparison of actual vs. Simulated market share for company 8

To verify the calibration accuracy of the agent-based simulation model, the Monte Carlo method was implemented for validation. Under this approach, the model is run under several random number seeds and averaged to give stable outputs. After calibration, the model was run for 1000 iterations to test its capability to mimic real-world dynamics of the Iranian pharmaceutical market. The validation results presented in Figs. 4, 5, 6, 7, 8, 9, 10 and 11 and Tables 10, 11, 12, 13, 14, 15, 16, 17, 18 and 19 compare actual versus simulated market shares for Companies 1–8 post-calibration.

**Table 10:** Comparison of actual vs. Simulated market share for company 1

Company 1	Actual	Simulation output (average)
2016	2.4%	2.4%
2017	2.6%	2.602%
2018	2.8%	2.801%
2019	2.8%	2.801%
2020	3.1%	3.069%
2021	3.2%	3.204%
2022	3.6%	3.604%
2023	3.7%	3.737%

**Table 11:** Comparison of actual vs. Simulated market share for company 2

Company 2	Actual	Simulation output (average)
2016	1.2%	1.2%
2017	1.4%	1.394%
2018	1.5%	1.504%
2019	1.5%	1.504%
2020	1.5%	1.504%
2021	1.5%	1.504%
2022	1.7%	1.722%
2023	1.8%	1.832%

**Table 12:** Comparison of actual vs. Simulated market share for company 3

Company 3	Actual	Simulation output (average)
2016	0.22%	0.22%
2017	0.3%	0.298%
2018	0.3%	0.298%
2019	0.3%	0.298%
2020	0.3%	0.298%
2021	0.3%	0.298%
2022	0.4%	0.391%
2023	0.4%	0.391%

**Table 13:** Comparison of actual vs. Simulated market share for company 4

Company 4	Actual	Simulation output (average)
2016	4.15%	4.15%
2017	4.4%	4.398%
2018	4.6%	4.596%
2019	4.7%	4.695%
2020	5.3%	5.295%
2021	5.3%	5.295%
2022	5.8%	5.792%
2023	6.1%	6.142%

**Table 14:** Comparison of actual vs. Simulated market share for company 5

Company 5	Actual	Simulation output (average)
2016	4.05%	4.05%
2017	4.4%	4.442%
2018	4.4%	4.442%
2019	4.8%	4.829%
2020	5.1%	5.123%
2021	5.1%	5.123%
2022	6.0%	5.999%
2023	6.3%	6.289%

**Table 15:** Comparison of actual vs. Simulated market share for company 6

Company 6	Actual	Simulation output (average)
2016	3.8%	3.8%
2017	3.8%	3.8%
2018	3.8%	3.8%
2019	4.0%	4.02%
2020	4.1%	4.093%
2021	5.4%	5.415%
2022	5.4%	5.415%
2023	5.4%	5.415%

**Table 16:** Comparison of actual vs. Simulated market share for company 7

Company 7	Actual	Simulation output (average)
2016	4.10%	4.1%
2017	4.0%	4.025%
2018	3.9%	3.879%
2019	3.7%	3.656%
2020	3.2%	3.288%
2021	3.1%	3.141%
2022	3.0%	2.992%
2023	2.7%	2.773%

**Table 17:** Comparison of actual vs. Simulated market share for company 8

Company 8	Actual	Simulation output (average)
2016	2.1%	2.11%
2017	2.1%	2.11%
2018	2.1%	2.11%
2019	1.9%	1.88%
2020	1.9%	1.88%
2021	1.5%	1.497%
2022	1.5%	1.497%
2023	1.5%	1.497%

With real market-share data taken into account, the model performance was further evaluated using the Monte Carlo average over 1000 replications with varied random seeds. The total deviation between simulated and observed market shares was kept within the bounds of 5% across all companies and years, while the variance of the Monte Carlo means had remained below 1%. Such low variance ensures model stability and convergence, adding great support to the position that it simulates real market behavior with negligible error.

## Numerical results

After calibration and validation, the simulation model was used to design and analyze different scenarios. The first scenario focuses on optimizing resource allocation between exploitation and exploration for the focal company agent. The second scenario performs sensitivity analysis to assess the impact of strategy improvement on market share. The third one considers how efficiently allocation is improved by learning from competitors’ experiences.

### Resource allocation optimization

Companies usually anticipate their expected future revenues which in turn determine resources available for exploration and exploitation. For this reason, a five-year forecast horizon was adopted for optimizing the allocation of resources to derive maximum market share. An optimization problem was thus formulated with the objective of maximizing market share under a constraint regarding the budget, which ensures that cumulative number of exploitation and exploration activities doesn’t exceed the annual budget.

12 $$Max Z = \mathop \sum \limits_{y = 2023}^{2028} Marketshar{e_y}$$

Subject to:

13 $$ExploitActivitie{s_y} + ExploreActivitie{s_y} \le Budge{t_y}$$

Budgets and costs associated with exploration and exploitation activities were estimated through expert interviews. The model initially assumes that expected marketing conditions remain stable over the next five years for the direct observation of internal strategic decisions, with external conditions persisting unchanged. This “stationary market” scenario provides the most apt benchmark by which to measure optimal resource allocation if the dynamics of the future replicate the past. Nevertheless, the framework is flexible, allowing other scenarios to deliberately vary marketing conditions, simulating different sets of competitive environment.

This optimization problem has been solved by simulating the optimization by Genetic Algorithm (GA), which has performed 5000 iterations and 1000 replications to ensure convergence and robustness. The optimized allocation for exploration and exploitation activities with expected market-share gains forecasted over a five-year horizon is elucidated in Table 18.

**Table 18:** Optimization result

	Over the past eight years			Optimized for the next five years		
	Type	${P_{exploit}}$	${P_{explore}}$	Type	${P_{exploit}}$	${P_{explore}}$
Agent Company	Analyzer	63%	37%	Prospector	0.25	0.75

**Fig. 12:**
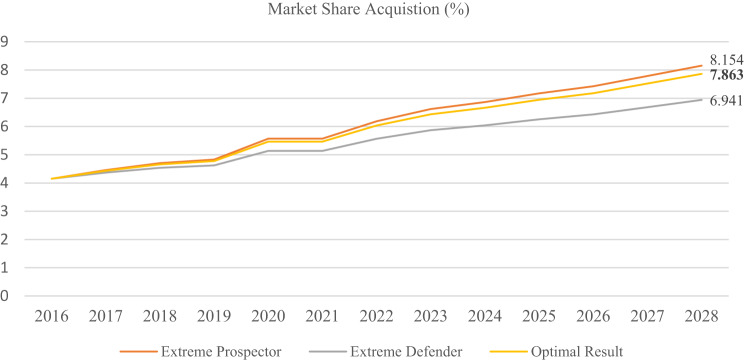
Market share acquisition by different company types

The optimized outcome suggests that the company agent is changing its configuration from an Analyzer to a Prospector, which in the current pharmaceutical scenario shows that, as compared to exploitation, exploring the market seems to bring greater market-share gains. These results will then be compared against other allocation options through a standard sensitivity analysis to ensure how relaxing the resource constraint affects optimal resource allocation. The less restrictive the constraint is, the more exploration the preferred optimal solution recommends, driving the strategic position of the firm toward an extreme Prospector. Two extreme benchmark scenarios were developed for comparison: Extreme Prospector, which guarantees best theoretical achievement under unlimited resource conditions, and Extreme Defender, which guarantees the worst minimal market share when only exploitation is appealed for. These benchmarks serve to compare the optimized outcome based on budgetary realities in terms of ideal and minimal possible performance levels. (Fig. 12). Results indicated that, under budget constraints, all configurations of Analyzer yield a lower outcome than the optimized one in terms of market share.

### Effective strategy implementation

The effectiveness of strategy implementation is another factor affecting the company agent’s market share. As previously shown, firms with lower implementation efficiency lost market share. To evaluate this effect, a sensitivity analysis was performed in which incremental improvements to the implementation of exploitation and exploration strategies were introduced into the agent-based model. Monte Carlo simulation with 10,000 iterations was subsequently employed to estimate the market share of the firm in the last year. The results are summarized in Table 19.

**Table 19:** Market share acquisition regarding improvement in strategy implementation

	Optimized	Market share (%) regarding improvement in strategy implementation						
		2%	4%	6%	8%	10%	15%	20%
The Agent Company	7.863	8	8.13	8.27	8.43	8.56	8.92	9.2

It shows, in addition to correct resource allocation to either exploitation or exploration methods, that the implementation of a strategy is critically important. Therefore, a proper resource allocation to the right strategy would still pose danger for an organization to reach an expected outcome due to an inappropriate implementation of the strategy.

### Improving resource allocation by leveraging the experience of other companies

**Fig. 13:**
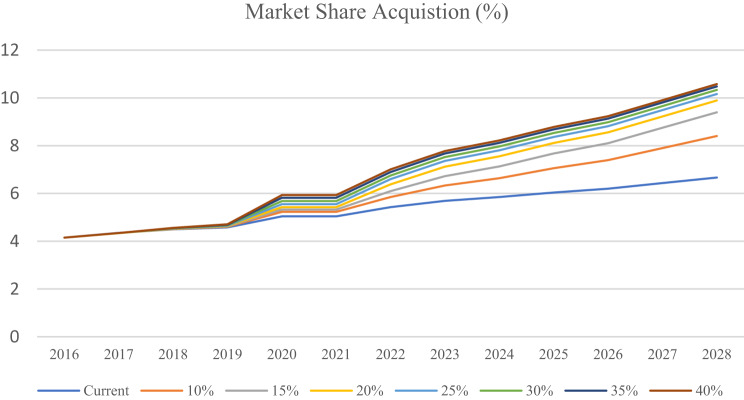
Sensitivity analysis of the market share achieved with different learning rates ($\alpha$)

In consideration of the dynamism in the pharmaceutical market, no one’s success can be sustained without continued adaptation of the resource allocation in exploration and exploitation. Resources can be reallocated based on learning from the competition by observing how they adjust their behavior in relation to market conditions. This process is defined by three parameters: D (information sample frequency), N (the number of competitors), and α (growth in learning). At each interval D, the company samples N competitors, evaluates their strategy outcomes, and shifts more of its allocation toward the better-performing strategy by α.With an optimistic evaluation of exploration market conditions (90% in favor of exploration vs. 10% for exploitation), sensitivity tests over varying D, N, α scenarios suggest the situation that maximizes market share occurs when the firm monitors every competitor (*N* = 7), collects information every year (D = 1), and applies the highest learning rate $\alpha = {\alpha _{max}}$. Resource reallocation is an area in which the market performance will benefit well from fast learning and timely action (Fig. 13).

**Fig. 14:**
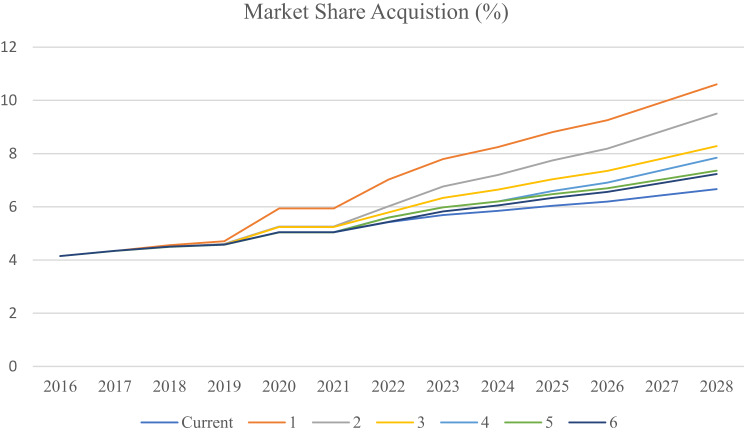
Sensitivity analysis of the market share achieved with different values of the learning interval (D)

Sensitivity analysis of a model for the market share shows that depending on a variety of competitor performance observation intervals (D), shorter updates for resource allocation to exploitation and exploration strategies tend to yield a larger market share. Notably, there is a remarkable difference in the growth of market shares between intervals of 1–3 years and the longer intervals (Fig. 14).

**Fig. 15:**
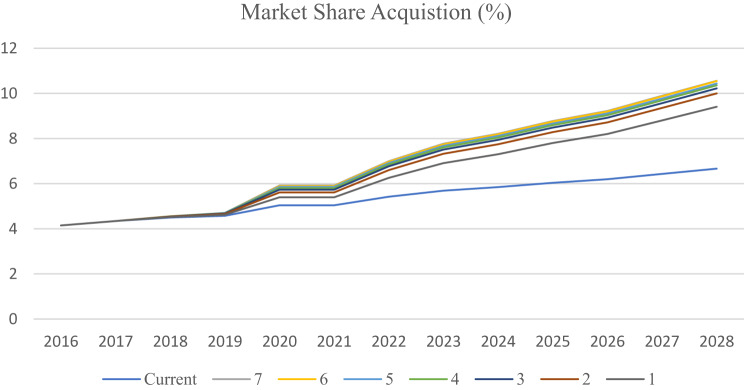
Sensitivity analysis of the market share achieved by learning from different numbers of competitors (N)

According to the sensitivity analysis of the simulation model (Fig. 15), the maximum market share scenario occurs when performance observations and resource allocation updates are assigned to more than one competitor. Market share remains stable when three or more competitors are analyzed.

In carrying out sensitivity tests, we could find that the learning rate (α), observation intervals (D), and number of competitors surveyed (N) influence the ultimate performance of firms to a fair extent, suggesting that certain of these parameters resemble actual managerial levers in these firms. The learning rate refers to the company’s ability to take in and utilize market intelligence either through analytical processes, training, or intelligence systems. The D variable indicates at what intervals strategic reviews are currently being held. Shorter time cycles (on a quarterly basis or so) would permit quicker adaptation. The number of competitors observed corresponds to the breadth of market scanning, which could be further widened through partnerships, collaborative research and development, or using open data platforms. Linking the above parameters to managerial practices thus translates simulation results to being actionable insights which could further strengthen adaptability and connect model outcomes to strategic decision-making processes.

## Discussion

The study focuses on the balancing act between the allocation of resources for the exploration of new opportunities and the exploitation of existing capabilities in the case of the Iranian pharmaceutical industry. Using Ambidexterity Theory, the Miles and Snow Typology, and Porter’s Five Forces, the study formulates an agent-based model that simulates strategic interaction between Company Agent, Competitor Agents, and Market Agent. The model was calibrated using Genetic Algorithms, and Monte Carlo simulations were subsequently used to validate it against data from eight major pharmaceutical companies from 2016 to 2023, thereby establishing a robust relationship with real-world dynamics.

The ABM framework illustrates how firms in the Iranian pharmaceutical market, shaped by Porter’s Five Forces, allocate their resources on a short-term focus on efficiency versus long-term focus on innovation. The findings show that Prospector-type firms, emphasizing exploration, witness higher growth in market share. Sensitivity analysis further indicated that easing budget constraints reinforces this outcome, as Prospectors outperform Analyzers under comparable conditions. This is especially salient with regulated markets in which the Ministry of Health (MOH) pricing schemes, calculated as the cost of goods plus a fixed margin, effectively ignore the R&D investments toward infrastructure, clinical trials, and long-term innovation. Such constraints can impose limitations on competitiveness and hamper the diffusion of new therapies [53]. If the incentives for exploratory investments—through targeted subsidies, tax incentives, or adjusted pricing under the MOH oversight—are introduced, then Iran’s pharmaceutical sector can become innovative and competitive, thus enabling companies to develop new therapies and enhance their market position.

This study methodologically progresses strategy modeling further than earlier analytical models or regression-based studies [10] without including behavioral adaptation into such models. Case-based research [54–56] has qualified insight but it never provided learning mechanisms formally. Although structural estimation is used to avail new data-driven approaches [57], it hardly does so in non-competitive environments. The present model fills this by embedding feedback loops in reproducing firms’ real adaptive behavior and market outcomes. Hence linking behavioral micro-foundations to empirical validation, it is a much sounder alternative to static or conceptual frameworks. This dynamic representation is consistent with recent evidence in organizational research, which shows that agent-based simulations more accurately capture adaptive and feedback-driven decision processes than static aggregation models [12].

## Conclusion

This study provides an evidence-based calibration of an agent-based model (ABM), optimized through genetic algorithms and validated through Monte Carlo simulation, and finds it to be robust in elucidating scenarios under which firms balance exploration and exploitation in their competition and institutional framing. The findings show that resource allocations leaning toward exploration can deliver stronger performance in volatile environments, and that adaptive, iterative learning processes help sustain long-term strategic flexibility.

In theory, this study brings together resource allocation theory, ambidexterity theory, and the Miles and Snow typology in a unified framework that conceptualizes ambidexterity as an emergent property of adaptive resource reconfiguration rather than a fixed structural choice. The research seeks to close a very important theoretical gap: how firms learn to reconfigure resources dynamically. It is no longer a question of mere description but a calibrated, behavioral explanation of strategic adaptation. Empirical calibration bridges conceptual strategy frameworks with observed decision patterns, broadening Bower’s view of resource allocation as the engine of strategic evolution.

Together with the incorporation of real data, those aspects will further qualify ABM beyond being merely a conceptual experiment, converting it into a validated decision-support tool in the spirit of new structural-estimation models such as those emerging in strategic management. The explanatory power of external validity combined with the strengthening of computational modeling in strategy research shall increase even further.

In practice, the findings underscore the importance of adaptive managerial mechanisms—such as flexible budgeting, continuous sensing of market signals, and targeted incentives for innovation—when navigating the trade-offs between efficiency and exploratory investment in regulated markets. Although the model draws its empirical base from Iran’s pharmaceutical sector, the methodological framework is applicable to other industries facing uncertainty and regulatory constraints. Also, the sole use of historical data between 2016 and 2023 would likely have hampered the prediction of disruptions triggered by technological changes or macroeconomic volatility. Behavioral factors such as managerial cognition, decision-making biases, and organizational culture have not been specifically modeled here, though they probably influence strategic outcomes and warrant more exploration.

Furthermore, an assumption of fixed budget was made that needs to lay bare internal trade-offs; future research could consider endogenizing budgets, tying them to performance and environmental feedback, thus reflecting better the adaptive financial decision-making. Future studies should introduce behavioral and policy variables to investigate how cognition, learning, and regulation shape dynamic resource allocation and ambidextrous performance across settings and industries. In addition, the empirical calibration relies on a homogeneity-based sample of very large pharmaceutical firms; consequently, firms outside the upper size quartile—such as smaller, family-owned, or less transparent companies—are not directly represented, and the findings should therefore be interpreted as analytically generalizable to structurally comparable firms rather than as population-wide statistical inference. Moreover, while policy effects are captured indirectly through competitive forces in the current model, future research may explicitly model regulatory shocks or alternative policy scenarios to examine their dynamic impact on firms’ strategic adaptation.

## Data Availability

The processed datasets generated and analyzed during the current study are available in the supplementary materials and can be shared with the journal upon request. Publicly available financial and pharmaceutical market data were obtained from Codal (https://codal.ir), the Iranian Food and Drug Administration (https://www.fda.gov.ir), and Data Pharma (http://www.datapharma.ir). Additional data collected from expert consultations and interviews with eight case study companies have been used to support the analysis.

## Abbreviations

ABM: Agent-Based Model
AHP: Analytic Hierarchy Process
GA: Genetic Algorithm
SSE: Sum of Squared Errors
MOH: Ministry of Health
IR: Intensity of Rivalry
TONE: Threat of New Entrants
TOS: Threat of Substitutes
BPOB: Bargaining Power of Buyers
BPOS: Bargaining Power of Suppliers
